# Parabens Accelerate Ovarian Dysfunction in a 4-Vinylcyclohexene Diepoxide-Induced Ovarian Failure Model

**DOI:** 10.3390/ijerph14020161

**Published:** 2017-02-08

**Authors:** Jae-Hwan Lee, Myeongho Lee, Changhwan Ahn, Hee Young Kang, Dinh Nam Tran, Eui-Bae Jeung

**Affiliations:** Laboratory of Veterinary Biochemistry and Molecular Biology, College of Veterinary Medicine, Chungbuk National University, Cheongju, Chungbuk 28644, Korea; phantom4015@nate.com (J.-H.L.); chris7spii@naver.com (M.L.); prac@naver.com (C.A.); nannaingir@hanmail.net (H.Y.K.); mr.tran90tb@gmail.com (D.N.T.)

**Keywords:** ovarian failure, paraben, folliculogenesis, steroidogenesis

## Abstract

Parabens are widely used preservatives in basic necessities such as cosmetic and pharmaceutical products. In previous studies, xenoestrogenic actions of parabens were reported in an immature rat model and a rat pituitary cell line (GH3 cells). The relationship between parabens and ovarian failure has not been described. In the present study, the influence of parabens on ovarian folliculogenesis and steroidogenesis was investigated. A disruptor of ovarian small pre-antral follicles, 4-vinylcyclohexene diepoxide (VCD, 40 mg/kg), was used to induce premature ovarian failure (POF). Methylparaben (MP, 100 mg/kg), propylparaben (PP, 100 mg/kg), and butylparaben (BP, 100 mg/kg) dissolved in corn oil were treated in female 8-week-old Sprague-Dawley rat for 5 weeks. Estrus cycle status was checked daily by vaginal smear test. Ovarian follicle development and steroid synthesis were investigated through real-time PCR and histological analyses. Diestrus phases in the VCD, PP, and BP groups were longer than that in the vehicle group. VCD significantly decreased mRNA level of folliculogenesis-related genes (*Foxl2*, *Kitl* and *Amh*). All parabens significantly increased the *Amh* mRNA level but unchanged *Foxl2* and *Kitlg* acting in primordial follicles. VCD and MP slightly increased *Star* and *Cyp11a1* levels, which are related to an initial step in steroidogenesis. VCD and parabens induced an increase in FSH levels in serum and significantly decreased the total number of follicles. Increased FSH implies impairment in ovarian function due to VCD or parabens. These results suggest that VCD may suppress both formation and development of follicles. In particular, combined administration of VCD and parabens accelerated inhibition of the follicle-developmental process through elevated AMH level in small antral follicles.

## 1. Introduction

In humans, premature ovarian failure (POF), also known as premature ovarian insufficiency [1], is characterized by amenorrhea, infertility, low estrogen levels, excess gonadotropin, and a lack of mature follicles before the age of 40 [2]. The occurrence of POF is mainly associated with the sustained reduction of the excess ovaries pool. Conventionally, in POF, the follicles do not develop properly. The exact mechanism producing inhibition of follicles in POF has not yet been described.

The chemical 4-vinylcyclohexene diepoxide (VCD) is known to induce POF in an experimental model [3,4]. The primary structure in the ovary is the follicle, which is composed of a germ cell (oocyte) surrounded by somatic cells (granulosa and theca interna) [3,5]. In mammals, oocytes are formed during fetal (human) or early postnatal (rodents) development from dividing oogonia, after which they become surrounded by a single layer of squamous-shaped pre-granulosa cells. This structure then forms the primordial follicle [6]. Because oocytes in the primordial follicle pool are arrested at the prophase of the first meiotic division, they are non-dividing and form the full cohort of germ cells that a female will possess throughout her lifespan. Thus, environmental chemicals with the potential to destroy the primordial follicle pool in women are of concern because exposure to those chemicals can lead to early menopause.

Endocrine disruptive chemicals (EDCs) can mimic hormones [7]. Such chemicals interfere with normal hormone function, resulting in negative effects on the regulation and reproductive mechanisms of the hormone [8]. Thus, evaluation of EDCs is important. Parabens are EDCs that are weakly estrogenic [9], and the strength of paraben’s toxicity depends on the length of its ester chain [10]. EDCs are primarily used as preservatives in consumer products including food, cosmetics, and pharmaceuticals. Parabens are rapidly absorbed through the skin and the digestive system and are then quickly degraded in the body. Biological and reproductive effects of parabens have been demonstrated through the expression of biomarkers of estrogen [11].

Early follicular development is regulated by paracrine and endocrine factors, including growth differentiation factor-9 (GDF-9), kit ligand/stem cell factor (KITL), anti-Mullerian hormone (AMH), and forkhead box protein l2 (FoxL2) transcription factor [9]. AMH is a peptide growth factor that belongs to the large transforming growth factor-b family, and is best known for its effects during sex differentiation, although it continues to be expressed in growing follicles in the ovary until they differentiate, at which point a dominant follicle is selected by action of the pituitary-derived, follicle-stimulating hormone [12]. AMH produced by mature follicles acts as an inhibitory signal that suppresses folliculogenesis of other immature follicles at the primordial-to-primary follicle transition. It has been reported that the AMH gene has functional estrogen responsive elements; therefore, its expression can be affected by estrogenic or anti-estrogenic compounds, including EDCs [13]. KITL, a growth factor in the ovarian follicle, is known to have a key role in mammalian embryonic and follicular development [14]. In vivo and in vitro studies have shown that KITL and its receptor, c-kit, participate in different stages of ovarian development, including the establishment of primordial germ cells, transformation of primordial follicles, oocyte survival and growth, proliferation of granulosa cells, recruitment of theca cells, and maintenance of meiotic competence [15,16]. FoxL2 is a member of the forkhead/HNF-3-related gene family of transcription factors, which are highly conserved and involved in many developmental processes, as well as in cellular differentiation [17].

Ovarian steroidogenesis is a complicated process involving multiple enzymes that together process cholesterol into biologically active steroid hormones. The steroidogenic acute regulatory (StAR) protein is a transport protein that controls cholesterol transfer within mitochondria, which is the rate-limiting step in the production of steroid hormones [18]. Cytochrome P450, family 11, subfamily A, polypeptide 1 (*Cyp11a1*) is a member of the cytochrome P450 superfamily of enzymes that catalyzes the first step of steroid hormone production [19], which leads to the conversion of cholesterol to pregnenolone at the inner mitochondrial membrane.

## 2. Materials and Methods

### 2.1. Chemicals

The 4-vinylcyclohexene diepoxide (VCD), methyl *p*-hydroxybenzoate (methylparaben; MP), propyl *p*-hydroxybenzoate (propylparaben; PP), and butyl *p*-hydroxybenzoate (butylparaben; BP) were obtained from Sigma-Aldrich (St. Louis, MO, USA). Stock solutions were diluted with corn oil (Sigma-Aldrich).

### 2.2. Animal Treatment

Eight-week-old Sprague-Dawley (SD) female rats were purchased from Samtaco (Osan, Gyeonggi, Korea). The animals were housed in polycarbonate cages in a controlled environment under an illumination schedule of 12 h light/12 h dark. Forty-eight female SD rats were divided into 8 groups of six rats each and were fed a purified diet (AIN-76A) and tap water. In addition, VCD (40 mg/kg/day) was administered intraperitoneally for 2 weeks. Subsequently, rats were orally administered MP, PP, or BP for 5 weeks. The concentrations of parabens required to produce estrogenic activity with no observed adverse effect levels (NOAEL) of each paraben were determined from those reported previously [20,21]. The each paraben was administered at a concentration of 100 mg/kg/day. All chemicals were dissolved in corn oil. The day after the last dose was administered, rats were euthanized via cervical dislocation. The ovaries, uterus, and blood were collected immediately. All animal experimental procedures were approved by Chungbuk National University Institutional Animal Care and Use Committee (IACUC) (project identification code: CBNUA-945-16-01).

### 2.3. Determination of Estrus Cycle

Vaginal smears were obtained to monitor the estrus cycle. Following collection of the smears by swapping vagina of rat, the morphology of the obtained epithelial cells were smeared and analyzed under light microscope to determine the number of cells at each of four stages due to the characters of each estrous cycle; proestrus, estrus, metestrus, and diestrus. The scores for each phase for each sample date were recorded.

### 2.4. Hematoxylin and Eosin Staining

Ovary tissues were fixed with 10% formalin. Subsequently, the tissues were embedded in paraffin and cut into sections (4 μm thick). To investigate the distribution of ovarian follicles in the ovaries, the prepared tissues were stained with hematoxylin and eosin (H&E), photographed, and scored in a blinded fashion by using light microscopy (BX51; Olympus, Tokyo, Japan). Images were captured by using an Olympus DP controller and manager at ×40, ×100, and ×200 magnification.

### 2.5. Quantitative Real-Time PCR

Ovaries were washed with cold, sterile saline and homogenized in TRIzol with a bullet blender (Next Advance, Averill Park, NY, USA). Total RNA was extracted from the homogenized solution by using TRI reagent (Ambion, Austin, TX, USA) according to the manufacturer’s protocols. Total RNA was measured by using an Epoch Microplate Spectrophotometer. Next, RNA (1 µg) was transcribed by using mMLV (Moloney murine leukemia virus) reverse transcriptase (iNtRON Bio, Gyeonggi-do, Korea) with a random 9-mer primer (TaKaRa Bio, Shiga, Japan) to produce first-strand complementary DNA (cDNA). The cDNA template (1 µL) was assayed by applying SYBR-ROX (TaKaRa Bio) real-time PCR according to the manufacturer’s protocols. Real-time PCR was performed under the following conditions: 40 cycles of denaturation at 95 °C for 30 s, annealing at 58 °C for 30 s, and extension at 72 °C for 30 s. Fluorescence intensity was measured at the end of the extension phase of each cycle. The threshold value for fluorescence intensity for all samples was set manually. The PCR cycle at which the fluorescence intensity threshold was in the exponential phase of amplification was designated as the threshold cycle (CT). The CT value was determined automatically at the exponential phase of the delta (Δ) CT fluorescence detection graph. The PCR product of RNA18S (18S ribosomal RNA) was used as an internal control for normalization. The amount of transcript present was inversely related to the observed CT, and the CT was expected to increase by one for every 2-fold dilution in the amount of transcript. Relative expression (R) was calculated using the equation R = $2^{-\left(\Delta \mathrm{CTsample}\;-\;\Delta \mathrm{CTcontrol}\right)}$. To determine a normalized arbitrary value for each gene, every data point was normalized to the control gene, as well as to the respective controls. Primer sequences used in quantitative real-time PCR are listed in Table 1.

### 2.6. Serum Hormone Analysis

Blood was collected from rats after the last chemical treatment. Serum was prepared immediately and stored at −70 °C until required for serum hormone analysis. A commercially available ELISA kit was used to measure the serum concentration of follicle-stimulating hormone (FSH; ELISA Kit, Cusabio, College Park, MD, USA). Quantitative determination of the hormone concentrations was performed according to the manufacturer’s protocols.

### 2.7. Data Analysis

Data were expressed as means ± standard deviations and were analyzed by using a nonparametric one-way ANOVA followed by the Tukey post hoc test (*n* = 6 per each group). All statistical analyses were performed by using SPSS for Windows (SPSS, Chicago, IL, USA). *p*-values <0.05 were considered statistically significant.

## 3. Results

### 3.1. Estrus Cycle Changes after Response to the Administration of 4-Vinylcyclohexene Diepoxide and Parabens

**Figure 1:**
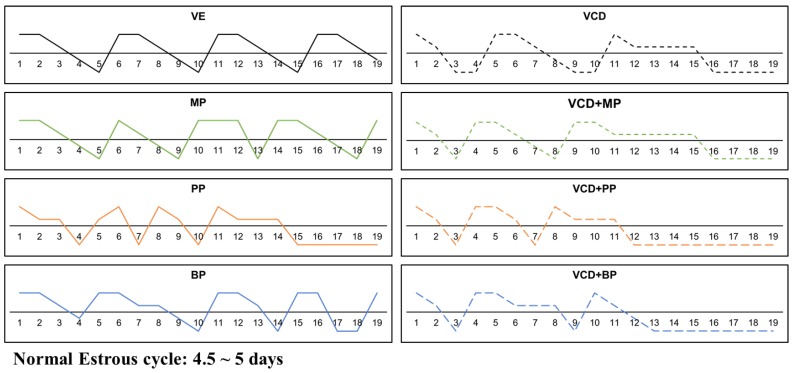
Effects of VCD and parabens on estrus cycles. The estrus cycle of the rats was monitored daily by vaginal smear test. The four cell types observed were scored and results presented graphically. Vehicle (VE) 20% Et-OH; VCD 40 mg/kg/day; MP, PP, BP 100 mg/kg/day.

During administration of VCD and parabens, estrus cycle status of 8-week-old SD rats was determined every day based on vaginal smear test results (Figure 1). The period of the rat estrus cycle is 4.5–5.5 days. The estrus cycle of the vehicle group showed a regular repeating cycle, while the VCD (16 days from start of administration) and VCD-paraben co-treatment groups (VCD + MP, 16 days from start of administration; VCD + PP, 12 days from start of administration; VCD + BP, 13 days from start of administration) and the PP group (15 days from start of administration) showed consistent diestrus. Combination with MP did not affect the time required to induce persistent diestrus, but BP and PP shortened this time. Unlike MP treatment, which showed a regular estrus cycle, treatment with BP and PP shortened the interval of the estrus cycle.

### 3.2. The Number and Development of Follicles Based on Histological Analysis

**Figure 2:**
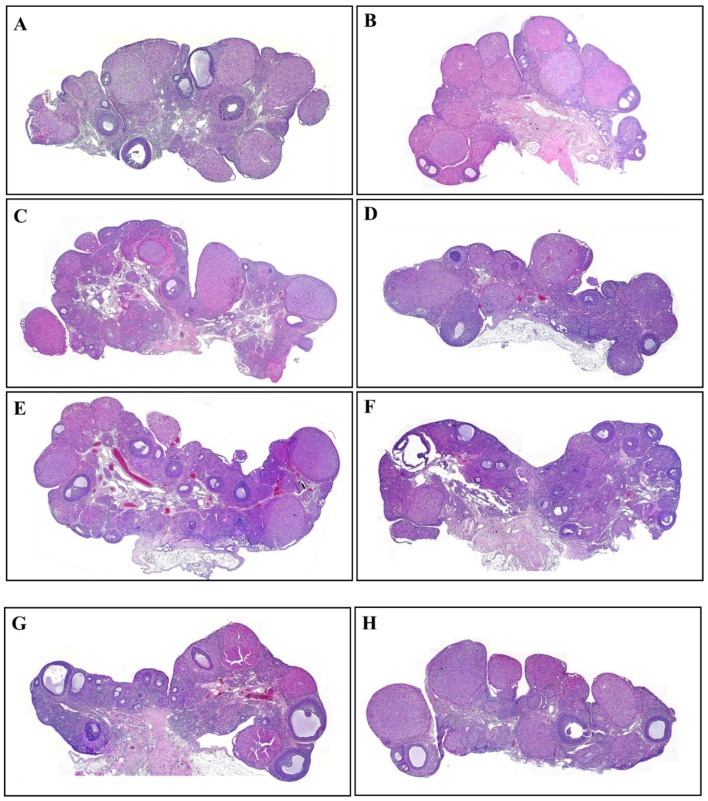
Effects of VCD and parabens on folliculogenesis in rat ovaries. The ovaries section were hematoxylin & eosin (H&E) stained for histological analysis. (**A**) vehicle; (**B**) VCD; (**C**) MP; (**D**) VCD + MP; (**E**) PP; (**F**) VCD + PP; (**G**) BP; (**H**) VCD + BP. VE 20% Et-OH, VCD 40 mg/kg/day, MP, PP, BP 100 mg/kg/day.

The ovaries extracted from rats were stained with H&E for histological analysis. Primary, secondary and preovulatory follicles and the corpus luteum were characterized based on the histological morphology of the granulosa cells and the oocytes. The follicles were counted and results compared among groups. Among the groups, there was no significant change in the number of these cells in the corpus luteum. However, treatment with VCD, parabens, and combinations of VCD and parabens resulted in a decrease in the number of total follicles (Figure 2).

**Figure 3:**
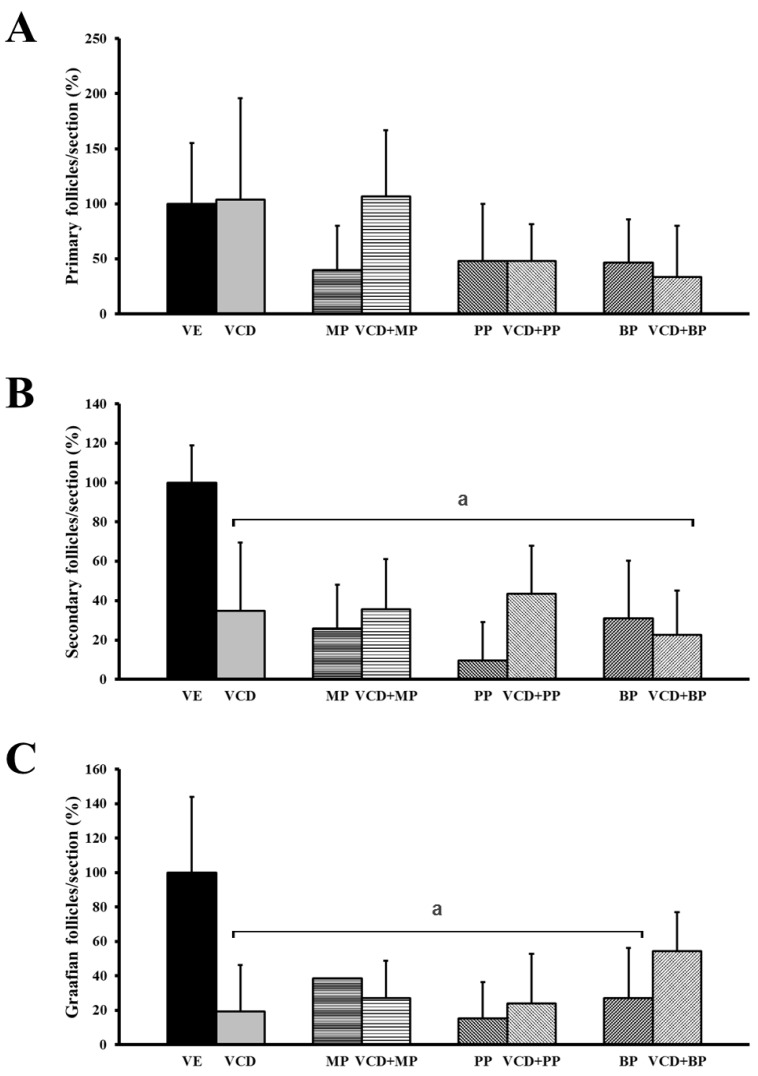
Effects of VCD and parabens on percentage of follicles at each follicle stage. Primary-, secondary- and preovulatory follicles were characterized by the histology of the ovary granulosa cells and oocyte morphology. (**A**) primary follicles; (**B**) Secondary follicles; (**C**) preovulatory follicles. ^a^ *p* < 0.05 vs. VE. Data are presented as the mean ± SD. VE 20% Et-OH, VCD 40 mg/kg/day, MP, PP, BP 100 mg/kg/day.

The number of primary follicles was not changed in the VCD or VCD + MP co-treatment groups (Figure 3A). Paraben-VCD co-treatment reduced the number of primary follicles, but the difference was not significant; however, paraben-VCD co-treatment did significantly reduce the number of secondary and preovulatory follicles from that in the vehicle group (Figure 3B,C).

### 3.3. Effects of Parabens on Premature Ovarian Failure Model of Follicle Development-Related Genes

**Figure 4:**
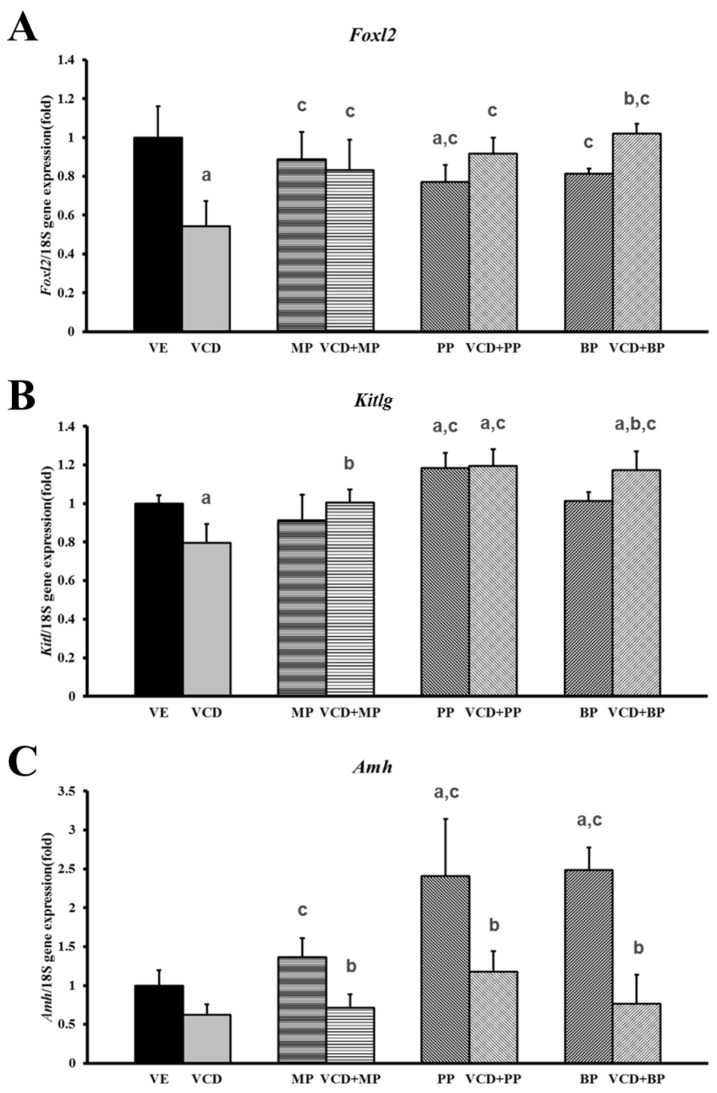
Effects of VCD and parabens on mRNA expression during follicle development in rats ovaries. The expressions of *Amh*, *Kitlg*, *Foxl2* gene (Table 1) were measured by real-time PCR and normalized by *Rn18s*. (**A**) *Foxl2*; (**B**) *Kitlg*; (**C**) *Amh*. ^a^ *p* < 0.05 vs. VE, ^b^ *p* < 0.05 vs. paraben, ^c^ *p* < 0.05 vs. VCD. Data are presented as the mean ± SD. VE; 20% Et-OH, VCD 40 mg/kg/day, MP, PP, BP; 100 mg/kg/day.

The mRNA expressions of the follicle development-related genes *Foxl2*, *Kitlg*, and *Amh* were measured by performing real-time PCR (Table 1). Expression of *Foxl2* mRNA level was downregulated by VCD, and recovery was only observed in response to administration of parabens and VCD-paraben co-treatment (Figure 4A). The level of expression was higher following co-treatment with VCD + BP than in the BP alone group. The expression of *Kitlg* mRNA decreased in response to VCD, but there was no effect in the MP and BP administration groups (Figure 4B). The VCD + MP and VCD + BP co-treatment groups showed increased mRNA expressions over those of the respective parabens alone groups. The PP, VCD + PP, VCD + BP treatment groups had higher levels of mRNA expression than that in the VCD group. The expression of *Amh* mRNA decreased in response to VCD treatment but increased in response to paraben treatments. Increasing levels of *Amh* expression were dependent on their toxicity (Figure 4C). The PP, BP alone treatment group showed a greater increase in expression than that in the MP group. The mRNA expression levels of all co-treated groups were reduced relative to their respective single paraben group.

### 3.4. Changes in mRNA Expression Levels in the Premature Ovarian Failure Model of Steroidogenesis-Related Genes

**Figure 5:**
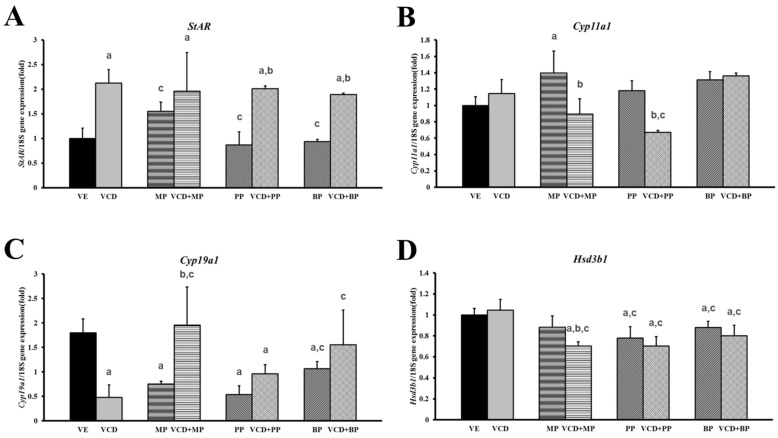
Effects of VCD and parabens on the mRNA expression of steroidogenic enzymes in rats ovaries. The expressions of *Star*, *Cyp11a1*, *Cyp19a1* and *Hsd3b1* gene were measured by real-time PCR and normalized by *Rn18s*. (**A**) *Star*; (**B**) *Cyp11a1*; (**C**) *Cyp19a1*; (**D**) *Hsd3b1*. ^a^ *p* < 0.05 vs. VE, ^b^ *p* < 0.05 vs. paraben, ^c^ *p* < 0.05 vs. VCD. Data are presented as the mean ± SD. VE 20% Et-OH, VCD 40 mg/kg/day, MP, PP, BP; 100 mg/kg/day.

The mRNA expressions of genes associated ovarian steroidogenesis (cholesterol-metabolism) such as *Star*, *Cyp11a1*, *Cyp19a1*, and *Hsd3b1* were measured by performing real-time PCR (Table 1). The expression of *Cyp11a1* mRNA was not altered by VCD treatment but was increased by MP treatment (Figure 5B). However, there was no increase in *Cyp11a1* in the VCD + MP co-treatment group. Treatment of individual parabens (MP, PP, BP) did not alter *Cyp11a1* expression, but VCD + PP co-treatment decreased *Cyp11a1* expression. The expression of *Cyp19a1* mRNA was decreased in the VCD, MP, PP, BP and VCD + PP treatment groups (Figure 5C). The VCD + PP and VCD + BP treatments led to increased mRNA expression relative to that in the VCD group. However, VCD + MP treatment had no effect on mRNA expression. The expression of *Hsd3b1* mRNA was not affected by VCD or MP alone, but VCD and MP, PP, and BP co-treatments and treatment with PP and BP alone led to decreased *Hsd3b1* expression (Figure 5D). Moreover, the VCD + MP treatment groups showed decreased expression compared to the VCD or MP alone groups. The expression of *Star* showed greater changes in response to treatment than that detected in the other steroidogenesis-related genes (Figure 5A). Specifically, VCD and paraben co-treatment groups’ mRNA expression levels were almost 2-fold higher than that of the vehicle group. The PP and BP administration group maintained expression levels similar to the vehicle group. In the parabens treatment group, the expression of *Star* mRNA was increased relative to the MP administration group.

### 3.5. mRNA Expression of Hormone Receptors Following VCD and Paraben Treatment

**Figure 6:**
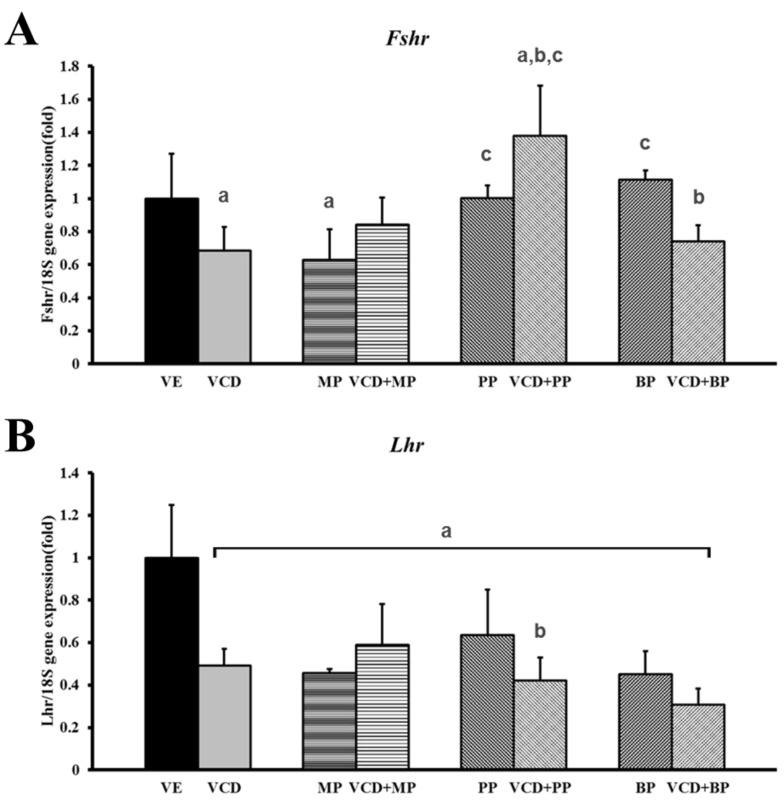
Effects of VCD and parabens on the mRNA expression of hormone receptors in rats ovaries. The expression of Fshr and Lhr gene were measured by performing real-time PCR, and normalized by *Rn18s*. (**A**) *Fshr*; (**B**) *Lhcgr*. ^a^ *p* < 0.05 vs. VE, ^b^ *p* < 0.05 vs. paraben, ^c^ *p* < 0.05 vs. VCD. Data are presented as the mean ± SD. VE; 20% Et-OH, VCD 40 mg/kg/day, MP, PP, BP; 100 mg/kg/day.

Expressions of the hormone receptor genes, luteinizing hormone receptor (*Lhcgr*) and follicle-stimulating hormone receptor (*Fshr*) were measured by performing real-time PCR (Table 1). *Fshr* mRNA expression decreased in the VCD and MP alone treatment groups. But the VCD + MP and VCD + BP co-treatment groups and the PP alone treatment group had no effect compared to the vehicle group. The highest expression was observed in response to VCD + PP co-treatment (Figure 6A). The *Lhcgr* expression level decreased significantly in response to VCD, parabens, and VCD-paraben co-treatments (Figure 6B). The expressions of *Lhcgr* mRNA in the parabens and co-treatment groups were similar to that in the VCD treatment group.

### 3.6. Follicle-Stimulating Hormone Levels in Each Experimental Group

**Figure 7:**
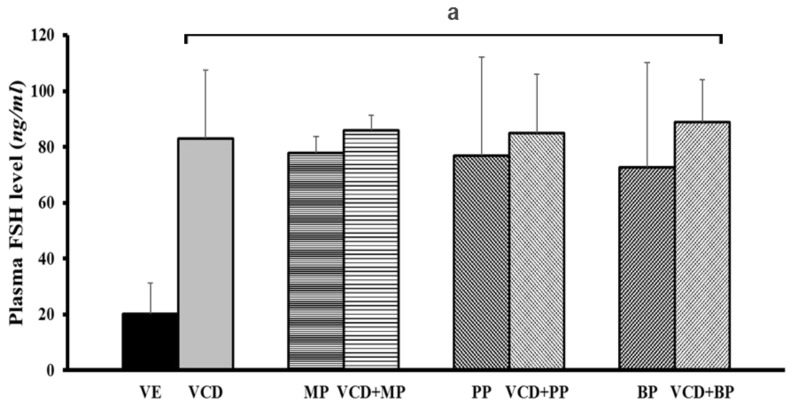
Effect of VCD and parabens on plasma FSH level. Plasma FHS hormone level were measured by ELISA. ^a^ *p* < 0.05 vs. VE. Data are presented as the mean ± SD. VE; 20% Et-OH, VCD 40 mg/kg/day, MP, PP, BP; 100 mg/kg/day.

The FSH level was determined through serum hormone analysis. The secretion of FSH increased in response to VCD, parabens, and co-treatments (Figure 7). The charateristics of premature ovarian failure is an increased FSH and decreased AMH level [22]. In the vehicle group, the FSH level was 20 ng/mL; however, in the VCD and parabens co-treatment groups this level was approximately 80 ng/mL. Overall, the significant increase in FSH in response to VCD, parabens, and VCD-paraben co-treatments indicates that ovarian failure is induced by VCD and/or parabens.

## 4. Discussion

Various EDCs are used for agricultural, medical, and daily purposes worldwide. When living organisms, including humans, are exposed to such compounds, the EDC can mimic endogenous hormones, thereby influencing regulatory systems within the organism. Simultaneous exposures to EDCs under a specific disease model that is related to hormones have been studied [23]. The present study was conducted to determine the effects of parabens in a VCD-induced ovarian failure model. Previous studies showed that VCD manipulated the time of onset of ovarian failure by accelerating the normal process of follicular atresia in rat [6]. Moreover, parabens mimic the actions of natural hormones and can stimulate or inhibit various enzymes required for hormone synthesis [21,24]. Recent studies have shown that EDCs such as parabens can modulate ovarian follicle development and steroidogenesis [9].

In this study, vaginal smears from female rats confirmed the presence of the four stages of the estrus cycle. The vehicle group consistently showed a normal estrus cycle. However, the VCD and VCD-paraben co-treatment groups showed either irregular estrus cycles or a continuous diestrus state. These phenomena are similar to symptoms in ovarian failure following damage of ovaries [24,25]. VCD has also been shown to selectively destroy oocytes in the ovaries of adults [26].

The development of follicles was evaluated to determine the effects of VCD and parabens. The numbers of secondary and preovulatory follicles were significantly reduced compared with the numbers in the VCD, paraben, and VCD-paraben co-treatment groups. VCD leads to depletion of follicles with destruction of small follicles [27]. In previous studies, PP and BP interrupted ovarian follicle development by increasing the number of primordial follicles and decreasing the early primary follicle numbers [9]. BPA treatment has delayed follicle formation in neonatal rat ovaries [13]. In addition, estrogen (E2) treatment has decreased the number of growing follicles [28].

During the biosynthesis of ovarian steroids, StAR mobilizes cholesterol into mitochondria, where it is converted to pregnenolone by CYP11A1. Pregnenolone is further metabolized by either CYP17A1 or HSD3B to produce dehydroepiandrosterone or progesterone [29]. Expressions of the steroidogenesis-related genes, *Star*, *Cyp11a1*, *Cyp19a1*, and *Hsd3b1*, were confirmed in our study. The mRNA expression of *Star* was unchanged in the paraben treatment group. However, expression of the *Star* mRNA was significantly upregulated by VCD, VCD + MP, VCD + PP, and VCD + BP treatments. Although there was no common effect among the tested parabens, it seems the effect of parabens on steroidogenesis expression differ with paraben structure. Only the MP group increased the expression of *Cyp11a1* mRNA. The mRNA expression of another steroidogenic marker, *Cyp19a1*, was significantly downregulated by treatment of VCD, MP, PP, and BP. These phenomena showed that parabens such as MP, PP and BP downregulated steroidogenesis. The mRNA expression of *Hsd3b1*, which plays a crucial role in the biosynthesis of all classes of hormonal steroids, was decreased by VCD + MP, PP, BP while MP or VCD alone did not downregulate the expression of *Hsd3b1*. Additionally, combination of VCD and PP, BP exerted negative effects on *Hsd3b1* expression. The results indicate that the parabens suppressed steroidogenesis by downregulating the steroidogenic enzymes. Moreover, VCD, which can mimic ovarian failure and VCD-paraben combinations, enhances the negative effect on steroidogenesis.

The early stages of folliculogenesis depend on gonadotropins and are regulated by interactions among paracrine factors, steroid hormones, and transcription factors [30]. In a previous study, Ahn et al. reported that parabens could interrupt follicular development [9]. In this study, we evaluated mRNA expression of various paracrine factors, including *Foxl2*, *Kitlg*, and *Amh*, to determine the state of folliculogenesis. KITL, a growth factor in the ovarian follicle, is known to play a key role in follicular development [31]. The results showed that VCD decreased *Kitlg* mRNA expression, while PP increased its expression. *Foxl2* may have a role in ovarian follicle maturation and can prevent premature follicle depletion, leading to POF [32]. The results showed that *Foxl2* mRNA expression decreased in response to VCD and PP. Parabens may regulate steroidogenesis by inhibiting follicular FoxL2, the main transcriptional repressor [9]. AMH is produced by granulosa cells in primary to small secondary ovarian follicles and helps maintain primordial follicles [33]. *Amh* mRNA expression did not significantly changed in response to VCD and increased in response to propyl paraben and butyl paraben. But in the co-treatment group showed decreased AMH levels compared to single paraben treatment groups. In previous studies, the mRNA expression of *Amh* was significantly increased in response to PP and BP (250 and 1000 mg/kg/day, respectively) treatments [9]. The effects of VCD on ovarian expression of *Amh* mRNA have also been investigated by performing time course analysis of PND4 rat ovary culture, and *Amh* mRNA was found to have decreased beginning on estrus cycle day four [33]. AMH is thought to inhibit primordial follicle recruitment, thereby facilitating the increase in primordial follicle recruitment caused by VCD [33].

Further, EDCs such as paraben can disrupt the functionality of antral follicles by decreasing ovarian mRNA, protein, and/or activity of the enzymes responsible for generating estradiol and its precursor sex steroid hormones [34]. EDC exposure caused the reduction in the expression of gonadotrophin receptors (*Lhr* and *Fshr*) [35]. Ovarian failure is defined by abnormally low levels of estrogen and high levels of FSH. To confirm the study’s VCD-induced ovarian failure model, serum FSH levels were examined. In the plasma treated with VCD, circulating levels of FSH were significantly elevated relative to that in control animals, indicating a loss of hormonal negative feedback to the pituitary from the ovary [36]. In our study, treatment with MP, PP, and BP alone or combination treatment with VCD and parabens elevated serum FSH levels. As antral follicles decreased, there was an associated increase in circulating FSH levels. These results were likely a result of a failure in the negative feedback associated with FSH release [37].

## 5. Conclusions

In conclusion, VCD- and paraben-induced POF in rat leads to the development of a depleted follicle supply by disrupting folliculogenesis and steroidogenesis. The result of that follicular depletion resembles the endocrine status and function of a postmenopausal ovary.

## Figures and Tables

**Table 1:** Oligonucleotide sequences for quantitative real-time PCR.

Species	Gene	Primer Sequence (5’→3’)	Accession Number	Product Size	Location of Primer Start
Rat	*Foxl2*	F: TCGCTAAGTTCCCGTTCTAC	XM_003750571.4	173	301
		R: GTAATTGCCCTTCTCGAACA			
	*Kitlg*	F: TTCAAGGACTTCATGGTGGC	NM_021843.4	164	451
		R: GCGGCTTTCCTATTACTGCT			
	*Amh*	F: CTGGCTGAAGTGATATGGGA	NM_012902.1	198	391
		R: CACAGTCAGCACCAAATAGC			
	*Star*	F: GCGGAACATGAAAGGACTGA	NM_031558.3	184	51
		R: TCCTTGCTGGATGTAGGACA			
	*Cyp11a1*	F: GCTTTGCCTTTGAGTCCATC	NM_017286.3	189	605
		R: CATGGTCCTTCCAGGTCTTA			
	*Cyp19a1*	F: GGCAAGCACTCCTTATCAAACC	NM_017085.2	197	671
		R: TCCACGTCTCTCAGCGAAAA			
	*Hsd3b1*	F: TGCCACTTGGTCACACTGTCA	NM_001007719.3	148	949
		R: CCCTGTGCTGCTCCACTAGTGT			
	*Fshr*	F: CTTGAAGCGGCAAATCTCTG	NM_199237.1	198	910
		R: GAGCAGGTCACATCAACAAC			
	*Lhcgr*	F: CTCACTGAAAACACTGCCCT	NM_001007719.3	198	817
		R: ATGGCGGAATAAAGCGTCTC			
	*Rn18s*	F: CTCAACACGGGAAACCTCAC	NR_046237.1	110	1251
		R: CGCTCCACCAACTAAGAACG			

F (forward); R (reverse).
